# Sociocultural perceptions of physical activity and dietary habits for hypertension control: voices from adults in a rural sub-district of South Africa

**DOI:** 10.1186/s12889-024-19320-0

**Published:** 2024-08-13

**Authors:** Kganetso Sekome, Francesc Xavier Gómez-Olivé, Lauren B. Sherar, Dale W. Esliger, Hellen Myezwa

**Affiliations:** 1 Department of physiotherapy, School of Therapeutic Sciences, Faculty of Health Sciences, University of the Witwatersrand, 7 York Road, Johannesburg, 2193 South Africa; 2 MRC/Wits Rural Public Health and Health Transitions Research Unit (Agincourt), School of Public Health, Faculty of Health Sciences, University of the Witwatersrand, Johannesburg, South Africa; 3 School of Sport, Exercise and Health Sciences, Loughborough University, Loughborough, United Kingdom; 4 School of Therapeutic Science, Faculty of Health Sciences, University of the Witwatersrand, Johannesburg, South Africa

**Keywords:** High blood pressure, Hypertension, Physical activity, Nutrition, Lifestyle change

## Abstract

**Background:**

Over half of adults from rural South Africa are hypertensive. Apart from pharmaceutical treatment, lifestyle changes such as increasing physical activity and reducing dietary salt have been strongly advocated for the control of hypertension. However, the control rates of hypertension for adults in rural South Africa are low. In this paper we explore whether this is due to the recommended lifestyle intervention not aligning with the individual’s socio-cultural determinants of behaviour change.

**Aim:**

To explore the social and cultural beliefs, perceptions and practices regarding physical activity and diet as a hypertension control intervention on hypertensive adults living in a rural sub-district in South Africa.

**Methods:**

Nine focus group discussions were conducted with hypertensive adults aged 40 years and above from Bushbuckridge sub-district in Mpumalanga Province of South Africa using a semi-structured interview guide. Each session began with introductions of the discussion theme followed by a short discussion on what the participants know about hypertension and the normal blood pressure readings. Physical activity and dietary habits were then introduced as the main subject of discussion. Probing questions were used to get more insight on a specific topic. A thematic analysis approach was used to generate codes, categories, and themes. A manual approach to data analysis was chosen and data obtained through transcripts were analysed inductively.

**Findings:**

Participants had a lack of knowledge about blood pressure normal values. Perceived causes of hypertension were alluded to psychosocial factors such as family and emotional-related issues. Physical activity practices were influenced by family and community members’ attitudes and gender roles. Factors which influenced dietary practices mainly involved affordability and availability of food. To control their hypertension, participants recommend eating certain foods, emotional control, taking medication, exercising, praying, correct food preparation, and performing house chores.

**Conclusion:**

Lifestyle interventions to control hypertension for adults in a rural South African setting using physical activity promotion and dietary control must consider the beliefs related to hypertension control of this population.

## Introduction

Hypertension is one of the world’s most prevalent and modifiable risk factors for cardiovascular and cerebrovascular diseases such as heart attacks and strokes [1–3]. Trends from 1975 to 2015 show that the number of people with hypertension worldwide has doubled from 594 million in 1975 to 1.13 billion in 2015 [2]. Reports suggest that 31.1% of adults worldwide had hypertension in 2010, with an estimated 9.4 million deaths (Mills et al., 2020;World Health Organization, 2013). Over the past decade, the prevalence of hypertension has increased worldwide, especially in low- and middle-income countries (LMICs). This reported prevalence was higher in LMICs (31.5%) than in high-income countries (28.5%) [4].

Hypertension or high blood pressure is defined as systolic blood pressure higher than 139 mmHg or diastolic blood pressure higher than 89 mmHg [2]. The most cited national prevalence of hypertension among older adults in South Africa is 77.3% in 2008 among a sample of adults aged 50 years or older [5]. In 2014, Lloyd-Sherlock and colleagues reported a South African hypertension prevalence of 77.9% amongst older adults aged 50 years and above [6]. These numbers can be attributed to factors such as ageing of the population, rapid urbanization, diet and lifestyle changes, and an increase in psychosocial issues [7]. The prevalence of hypertension in rural South Africa for individuals aged 15 years and above is reported to be 41% in Limpopo (mean age = 44.2 ± 20.9) (Dikgale Health and Demographic Surveillance Site) [8] and 40% (women), 30% (men) in Mpumalanga (mean age not reported) (Agincourt Health and Demographic Surveillance Site) [9]. Some of the reported risk factors for hypertension in LMICs are overweight/obesity, being married, older age group, low education, and alcohol consumption [7, 10–12].

People 40 years and older in rural Mpumalanga seem to be aware of their blood pressure status (awareness rate − 64.4%) and amongst those aware most are on medication for hypertension (89.3%) although the level of control remains low (45.8%). However, the number of people receiving treatment dropped to 49.7% when considering the overall hypertensive population and not only those who were aware of the condition [13].

A longitudinal observational study in rural Agincourt that followed individual adults on usual care for hypertension, found improved rates of hypertension over a four-year follow-up period [14]. Studies for hypertension control using physical activity in South Africa focused on monitoring and goal setting (increasing the number of steps taken a day) [15, 16]. Studies targeting dietary changes for hypertension control have focused on providing advice on diet that is usually not affordable or not accepted by the rural adults [17, 18]. There is one known study in rural Agincourt of South Africa aimed to reduce hypertension through provision of monthly supply of low sodium salt [19], where participants expressed a challenge with sustainability of this approach because the sodium salt product is not available in local stores. For effective implementation of this recommendation, one must first understand the contextual (personal and environmental) factors regarding salt and calorie intake in this population.

Providing a description of what individual factors contributed to the improved hypertension control rates [14], considering the influences of physical activity performance [16], and understanding the socioeconomic factors contributing to dietary consumption [17, 18] could yield better hypertension control rates. A recent systematic review concluded that future research in developing countries should consider individual risk perceptions, cultural barriers, and gender when designing interventions for hypertension control [20].

Various personal and/or environmental factors along with the person’s level of functioning can influence the uptake of an intervention [21]. For example, physical activity interventions to control hypertension in rural South Africa may be different from other populations more studied due to the daily physical activities in rural South Africa such as walking for wood collection, different aspects of farming, yard work, walking longer distances for daily chores, and housework [15, 22]. Activities are also likely constantly evolving because of urbanization, migration, and an ageing population [7, 23].

When developing interventions in South Africa to reduce hypertension, there is a need to consider the cultural and social beliefs and practices that influence behaviour of the rural adult population. Both a reduction in salt and caloric intake, and an increase in physical activity are the most extensively recommended lifestyle approaches to help meet national hypertension targets [24–26]. However, promoting change in behaviour of someone through an intervention needs an understanding of the contextual realities at play that influence that person’s behaviour. We aimed to explore the social and cultural beliefs, perceptions and practices regarding physical activity and diet from hypertensive adults living in rural South Africa and to explore how these perceptions may influence future behaviour change. This should inform novel interventions for the control of hypertension in this population.

## Methods

### Setting

This qualitative study was conducted at Bushbuckridge sub-district in Mpumalanga province South Africa where the Agincourt Health and Demographic Surveillance System (HDSS) has been running since 1992. The HDSS is run by the MRC/ Wits Rural Public Health and Health Transitions Research Unit from the University of the Witwatersrand. The Agincourt HDSS covers an area of 450km^2^ with 115 000 individuals (approximately 52 000 older than 18 years) living in approximately 20 000 households [27]. We used as a sampling frame the cohort of 5059 adults aged 40 years and above within the Agincourt HDSS, named the Health and Aging in Africa: A Longitudinal Study of an INDEPTH (International Network for the Demographic Evaluation of Populations and Their Health) community in South Africa (HAALSI) [28]. The prevalence of hypertension in this cohort is 57%, defined as systolic blood pressure ≥ 140 mmHg or diastolic blood pressure ≥ 90 mmHg or self-reported medication of hypertension.

Healthier food options in this setting are available in large supermarkets, but due to the high costs involved people often grow their own food [17]. Cheaper food items are available in smaller food stores, but availability of healthier food is limited. Physical activities are mainly centred around the performance of activities of daily living such as walking, livelihood activities, and household duties [22].

### Population selection

Participants for the focus groups were randomly selected from the HAALSI cohort including men and women aged 40 years and above with a diagnosis of hypertension. We chose a random sample because we wanted to observe opinions from a mixed sample (age, gender, awareness of hypertension status). Participants must have been living in Agincourt for the past six months prior to the start of the focus group discussions to ensure that they have adapted to the daily routine of the community. We produced a random list of 150 potential participants, and they were contacted by phone and asked to participate in a focus group on high blood pressure. Participants were interviewed until data saturation required was reached. Participants were contacted by a locally trained research assistant who speaks the local language (Xitsonga), and they were compensated for their participation in the focus group discussion.

### Focus group preparation

The focus group interview guide (Appendix A) was developed by the first author and reviewed and approved by all co-authors. The focus group guide included questions on physical activity and dietary practices and influences, barriers and enablers of physical activity and dietary approaches, for hypertension control.

### Focus group discussions (FGD)

FGDs were facilitated by a female locally trained qualitative research assistant. Moreover, the first author and a second male locally trained qualitative research assistant were present to co-facilitate. Each FGD included 6 to 9 participants and lasted 45 to 90 min. Each session began with introduction of the discussion theme and then followed by a short discussion on what the participants knew about hypertension, the average normal blood pressure reading as well as their awareness of their blood pressure status. Physical activity and dietary habits were then introduced as the main subject of discussion using the focus group guide. Probing questions were introduced to get more insight on specific topics if needed. The FGDs were audio recorded and the co-facilitator documented the discussion during each session. Translation to English and transcription of the audio recorded interviews occurred after each session. The FGD were performed at a private venue in the Agincourt HDSS premises. All participants were fetched from their homes and dropped off after the session.

### Trustworthiness of data

We ensured trustworthiness of our data by following the principles of credibility, transferability, dependability, confirmability, and reflexivity [29]. To ensure credibility of the data, locally trained qualitative research assistants were employed to conduct the FGDs and transcribe the data. The transcribed FGDs audio data was reviewed by the first author and all co-authors. The first author received feedback from all co-authors on the interview guide ahead of data collection and amendments made when indicated. Throughout the data analysis process, codes were generated and reviewed by three experienced peer qualitative researchers not involved in the research project as well as a senior co-author (HM). Transferability, though limited in qualitative research, was ensured by providing a thick description of the study setting and population selection in this paper to allow for repeatability. Dependability was achieved through inviting qualitative researchers not involved in the research study to view and give feedback on the field notes and initial codes created through a debriefing session. The authors have also kept an audit trail of interview recordings and transcripts. The first author kept a clear focus during the data analysis phase by examining his own judgments, practices, and belief systems regarding the topic of analysis through a reflexive journal which documented the study rationale and methodological decisions. To ensure confirmability, direct excerpts from the interviews are provided under the findings section.

### Data analysis

A thematic analysis approach was used to generate codes, themes and categories. All transcripts were analysed using an inductive approach. We employed a manual analysis technique to generate codes, categories, and themes. This process included printing hard copy transcripts, photocopying, line-by-line coding, coding in margins, cutting, cut-and-paste, sorting, reorganising, hanging and arranging colour-coded sticky notes on a large display wall [30–32]. The five-step approach to qualitative data analysis outlined by [33] was followed.

### Findings

The analysis of the data revealed eight main themes: (i) lack of knowledge of normal blood pressure values; (ii) perceived causes of hypertension; (iii) variety in daily physical activity practices; (iv) limited variety in daily diet; (v) ways in which the participants control their hypertension; (vi) alternative beliefs for hypertension control; (vii) factors that influence their dietary practices; and (viii) community influences on physical activity. The themes are presented with their categories and accompanying excerpts from the participants (Table 1). The daily dietary practices are presented on a separate table to indicate the frequency of consumption (Table 2).

**Table 1:** Findings from focus group discussions according to identified themes, categories, codes, and participants excerpts

Theme	Category description	Identified codes	Participant excerpt
Lack of knowledge of normal blood pressure values.	Lack of knowledge about normal BP values. Field workers from the studies run by MRC/Wits Agincourt Research Unit had some influence on their knowledge of normal BP values.	No feedback Fieldworker influence	*“They (nurses) don’t tell us the numbers they only tell us that it is normal or high”. FGD7, P2.* *“They (Agincourt HDSS field workers) do show you the numbers but at the clinic they don’t show you”. FGD5, P5.*
Perceived causes of hypertension.	Family dynamics. Emotional causes. Poor eating habits.	Spousal induced stress, stress caused by children, partner abuse, family conflicts, and family loss due to death. Loneliness, anger, sadness, and worrying too much as the main contributing factors. Eating sweet food items, an urbanised diet, cold water consumption, oily and fatty diet, salty and spicy diet, and obesity.	*“Sometimes you find that at home you have a husband and is not treating you very well, even if you can hide it but deep inside from your heart you are worried. We also have children, and they are speaking to us with bad manners, so even if we run away from high blood pressure in that way you can find yourself with high blood pressure”. FGD1, P8.* *“it’s caused by thinking a lot, because when you worry too much each and every day about things that is troubling you, and when you worry too much it becomes to the point where your life begins to change and being diagnosed with high blood pressure”. FGD1, P8.*
			*“The food that we eat now is the one which causes us high blood but before we used to eat traditional food and some of those food was very sour and that help us to be healthy. But now we eat modern and some of those food is not good for our health”*. *FGD3, P6.*
Variety in physical activity practices	Sedentary activities. Social and cultural activities. Work/livelihood activities. Household activities. Purposeful exercise. Routine activities. Walking activities. Unstructured physical activities.	Sewing mat, grinding maize, and ploughing while seated. Dancing at church and teaching traditional dancing. Selling at a tuckshop, shop cleaner, sweeping the yard, construction of bricks, cutting poles at the forest, farming work, domestic worker, and ploughing. Wash dishes, cook daily, yard work, and taking care of family. Morning jog, jogging after work, a daily home exercise program, female soccer player, running at soccer field, and stretching daily. Fetching firewood from the bush, gardening, running around with children, physical activity health educator, daily ploughing, fetching water with wheelbarrow, and cleaning the yard. Herding cows, walking to the bush, walking in the neighbourhood. Based on daily household needs and general housework	*“I don’t know how to run, I plough while I am sitting on the chair, I grind maize meal while I am sitting on the chair”. FGD4, P1.* *“I wash clothes or clean the house…. then after I come sweep the yard and make some fire and cook, then I go to work”. FGD4, P6.* *“When I wake up in the morning before I do anything I start by exercising inside the house”. FGD4, P8.* *“We teach the elderly people to do physical activities, such as stretching, exercising, playing soccer, and we also practice running with them”. FGD1, P8.* *“…and then I go outside and sweep the yard. Sometimes I play with my grandchildren, running around with them”. FGD4, P3.* *“You have to do activities like walking around then your blood pressure will be reduced. If you sit in one place without moving your blood pressure will rise”. FGD1, P5.* *“When I wake up, I just go around my yard and see what I can do, I clean the yard removing grass, fixing some broken fence. Then after that I rest.” FGD9, P6.*
Ways to control hypertension	Emotional control. Medical control. Structured exercise routine. Dietary control. Involvement in house chores.	Social interactions, reducing stress, avoiding anger, avoiding over-excitement, accepting situations, avoiding stress, and finding ways to manage anger. Follow the prescribed treatment regime, follow health worker advice, adhere to medication, adhere to doctor’s diet advice, avoid medical pluralism, and practice early health seeking behaviour. Perform daily exercises, have a daily exercise plan, join a female soccer club, go for a daily jog, increase levels of physical activity at home, increase walking distance, walk daily. Drink tea instead of coffee, avoid spicy diet, avoid salt and spices, avoid oil and fats, increase fruits and vegetables, eat more bitter vegetables, drink water, prepare own meal, avoid sugar, stop spreading bread with margarine, and grow own vegetable. Increasing gardening work, increasing chores which require exercise, performing daily house chores, and increasing pace of tasks.	*“because of our age we think a lot about death, but if you meet other people like as we are here today, we are busy chatting about lot of things and some they do jokes, and we laugh”. FGD1, P8.* *“If you are hypertensive don’t mix medication, if you are taking high blood pills take them only, don’t mix it with traditional medication because the high blood pressure won’t be controlled”. FGD1, P5.* *“The only thing that can reduce high blood is to eat Muroho and Nkaka. Nkaka it can reduce the high blood pressure. And also cabbage, if you can get used to chop the cabbage and not add salt just eat it as it is raw, the high blood pressure will be reduced”. FGD1, P5*
Alternative beliefs for hypertension control.	Spiritual means. Ways of preparing food. Alternative food to consume.	Prayer, denial and avoidance of diagnosis, and accepting situations. Drinking boiled marijuana, drinking aloe boiled in water, drinking warm water with sugar, and drinking water of boiled local trees. Raw potato, moringa leaves, moringa juice, and Nkaka.	*“Another thing is to accept difficult situation that can cause high blood pressure. Sometimes you have to pray and ask God, you become better”. FGD4, P5.* *“Indeed, there’s this tree that is called Moringa I know it, and even Nkaka I eat it. When I use these things, I can see the difference. Even when I go to the clinic the nurses ask me ‘what are you using?’”. FGD9, P6.* *“The other thing that I heard about it is Moringa powder and Moringa juice they said it is helpful to reduce high blood pressure”. FGD3, P9.*
Factors influencing dietary practices	Lack of diet control. Food taste. Food safety, affordability, and availability.	Eating outside home (such as at a party or funeral), community food project provision, religious influence, not preparing own food, children preference, and donated food. Fatty and oily food preferred because it’s delicious, Rama margarine makes food taste good, craving for spicy food, “hypertensive” meals are not enjoyable, and participants love bitter vegetables. Most vegetables are difficult to access, participant eats whatever is available, there is a lack of money for variety, and the prescribed diet is expensive.	*“Sometimes you find out that you are not at home you visited somewhere and because of hunger you end up eating food that you were not supposed to eat”. FGD1, P3.* *“Another thing is because of some of the food they just buy and give us, when they give you food you won’t refuse knowing that there’s nowhere you can get food”. FGD1, P6.* *“Those who are hypertensive you won’t like their food because they don’t put salt at all, they just eat like that because they are hypertensive. They don’t put oil in their food because they are hypertensive”. FGD1, P1.* *“I can say the food that I am eating at home is the food that I am affording.” FGD1, P8.* *“Another thing at our church we don’t eat lot of things that’s the reason why I am not eating some of the things. If it was not for my church, I would eat anything”. FGD8, P4.*
Influences of community on physical activity.	Influence of family. Physical activity seen as a must. Disregarding opinions from the community.	Children see physical activity as punishment and complain that the parent ploughs too much, family viewed the parent as working a lot, children question the reason for daily chores, physical activity tasks were seen as suffering, parents are told by children to sit and rest. Daily chores are forced by situation, being head of the household, and the chores need to be done. Neighbours viewed physically active people as working too hard.	*“They are like: what kind of a woman are you always doing the house chores?” FGD1, P1.* *“Because you are the man of the house there’s no way you can rest, a man does not rest”. FGD1, P6.* *“So, I told them who is going to do the work for me because I do this and this, if they (children) work, they do little things and rest”. FGD5, P6* *“Others will say that I love working too much so you don’t have to mind them because it can cause you high blood if you want to listen to them” FGD2, P4*

### Limited variety in daily diet

The daily dietary practices were categorised into breakfast meals, lunch meals, and dinner meals. These meals are presented in Table 2 together with the frequency in which they are consumed. All meal items are consumed for breakfast, lunch meals consist of bread, porridge, soft porridge, vegetables, meat, and tea. For dinner the consumed food items are porridge, vegetables, meat, and tea.

**Table 2:** Daily meals consumed by hypertensive participants

Meal item	Breakfast	Lunch	Dinner
Bread	✓	✓	✓
Porridge	✓	✓	✓
Soft porridge	✓	✓	-
Mageu & Amasi (fermented porridge & milk)	✓	-	-
Vegetables	✓	✓	✓
Wild vegetables	✓	-	✓
Meat	✓	✓	✓
Tea	✓	✓	✓

## Discussion

This qualitative study identified eight major themes relating to hypertensive adults’ lack of knowledge of normal blood pressure values, perceived causes of hypertension, variety of PA practices, ways to control hypertension, alternative beliefs for HPT control, factors influencing dietary practices, the influence of community on PA, and limited variety in daily diet. These insights are key to guide interventions on sociocultural considerations when developing strategies to control hypertension from a rural, African lens. Few studies have reported on hypertensive adults’ knowledge of normal blood pressure values; however, some studies have investigated knowledge about hypertension as a health condition [34–38]. Our study found that hypertensive adults have little to no knowledge about high blood pressure normal values, the little knowledge which they have is obtained from ongoing research studies conducted in their community [27]. A study in Ghana by Agyei-Baffour [39] also reported that knowledge of hypertension was very low in their participants. They reported that those participants who had knowledge about hypertension obtained it from the media, followed by health staff. Our study showed that health staff have very little influence on hypertension education and sharing feedback on measured blood pressure results in this community. Suggestions for health promotion in rural communities from previous studies have included alternates to clinic-based education, such as door-to-door, peer education (teaching the youth who will teach the adult), working with local church leaders to provide church-based education, and providing education via pamphlet handout and billboards [37].

In almost all our focus group discussions, participants spoke about the influence of poor emotional wellbeing and stress-related factors as causes of elevated blood pressure. They referred strongly to stress induced by family members. This finding is not unique to our study and has been reported by participants in rural KwaZulu-Natal who described that unhappiness and stress were major contributing factors to elevated blood pressure [40]. Similarly, Jongen and colleagues [37] identified stress/anxiety as factors that prompt the development of hypertension. The participants in their study [37] reported that fighting with children or spouse or the inability to support the family financially due to unemployment were the major triggers of stress and/or anxiety. There appears to be an unmet need for psychosocial support in rural South Africa.

Regarding the influence of diet as a cause of hypertension, our study participants recognised that high sodium salt consumption, oily and fatty food items, and certain spices can cause hypertension. These risk factors have been reported and confirmed by other studies as contributors to the development of hypertension [37, 41, 42]. Participants in our study also identified being obese as a cause of hypertension. There is a reported positive relationship between obesity and overweight with hypertension [43, 44].

Our results highlight the variety of sociocultural and livelihood physical activities adults perform as part of their daily living in this setting. These activities include walking while herding cows, sweeping the yard, ploughing, and walking to fetch firewood which all have been previously reported by other studies as playing a major part of physical activity involvement for rural adults [45–47]. It is important to note that most of these reported activities, such as sewing mat while seated, household activities, walking activities, and unstructured activities are defined as sedentary or light/moderate intensity physical activity [46]. Public health interventions aiming at increasing physical activity for hypertensive adults in a rural population may therefore want to target increasing the intensity of already existing physical activity practices.

Family members of hypertensive adults appeared to often discourage physical activities of high intensities. The participants are told by their family members that doing chores every day is a form of punishment and suffering. However, the participants continue performing these activities due to lack of help. This suggests that most house chores in rural South Africa are left for the adults, an opportunity which can enhance physical activity participation for health benefits. Indeed, participants recognised that performing daily chores was beneficial to their health because it keeps their heart pumping more blood and this is good to control their blood pressure. It was interesting to note that females are often discouraged from performing chores linked to vigorous intensity physical activities instead of the males. This has been explained by Oyewumi [45] who highlighted the role of gender in household activities. The author explains that adult men are expected to perform more vigorous intensity physical activities at home such as heavy lifting, chopping wood, or digging in the garden and women are expected to perform activities of lower intensity. The influence of gender cannot be ignored when designing public health physical activity interventions for blood pressure control.

Dietary approaches to stop hypertension (DASH) is a globally recommended approach to dietary habits for hypertension control which has been in existence for over 25 years [48]. The DASH diet is considered the golden standard in high blood pressure control, and it emphasizes foods rich in potassium, protein, fiber, magnesium, and calcium [49]. Such foods may include fruits and vegetables, nuts, beans, whole grains, low-fat dairy, and limiting food high in saturated fat. However, the daily diet consumed by participants in our study indicates lack of variety of different food types. Participant’s diet appears mainly rich in starch which is high in carbohydrates as seen by the bread, porridge, soft porridge, and fermented porridge and milk consumed daily.

Our study revealed multiple factors which are linked to the reasons why hypertensive adults in our study setting may not follow the DASH diet recommendations. Lack of control in the type of food eaten because of attending social events like parties and funerals, where there is no choice of food, was a contributing factor. In rural African settings, when attending a social event, it is uncommon to have different meals prepared based on an attendee’s dietary recommendations. Although social and community gatherings happen occasionally in rural South African populations, the consumption of unhealthy diet such as high dietary sodium in the long-term can lead to dysregulation of the renin-angiotensin system, contributing to hypertension [50]. Religion was another influence in our study where certain religious groups prohibit eating certain types of food and may promote other types of food and beverages regardless of an individual’s health status [51]. In addition, participants reported that the taste of the food played a major role in food choice. They found that unhealthy food items taste better and reported that “hypertensive meals are not enjoyable”. Another important contributor to food choice mentioned by our participants and that has been reported in multiple studies was affordability and availability of different, healthier foods [17, 52, 53].

Due to the high poverty and unemployment rate in rural areas of South Africa, people often eat what is affordable and readily available [54]. Most families living in poverty purchase larger quantities of less healthy food products compared to smaller healthier substitutes [37]. This highlights the importance of considering the local financial context when designing dietary interventions for the control of elevated blood pressure. Charlton and colleagues [55] explain that sometimes the DASH diet is not realistic to people living in rural areas with high unemployment and poverty. They recommend that the best dietary approach to controlling hypertension may be making small adjustments to current daily salt intake instead of advising people to change what they eat. This recommendation is important to consider as it has proven effective in other similar settings [24, 56, 57]. In our study setting, it was recently reported that 65% of study participants aged 40–75 years had a mean salt intake of 6 g/day [58]. This is higher than the recommended daily salt intake of 5 g/day by World Health Organization [59].

Participants in our study were knowledgeable with regards to strategies for controlling hypertension. They mentioned the importance of psychosocial wellbeing, adhering to pharmaceutical treatment, dietary control, and involvement in daily household chores and exercise. These measures have been well documented in literature as effective strategies for hypertension control [60–62]. Regarding psychosocial wellbeing, our participants highlight the value of engaging with other people and sharing positive thoughts and having a spiritual connection with higher powers. They expressed that managing stress and anger is important in controlling hypertension. These findings have also been reported by adults in a study by Jongen [37] and in a church-based intervention by Tussing-Humphries [63].

Participants in our study believed that traditional medicine must not be mixed with pharmaceutical/western medicine. This may be attributed to the ongoing research and collaborations between traditional healers in the communities and the research team (WITS HDSS Research and Surveillance System). One important wild herb that adults in our study setting strongly emphasised as assisting in high blood pressure control is *Nkaka* (Momordica balsamina), also known as the African Pumpkin. Although it is not a cure for hypertension, this herb is reported to contain high potassium content which is good for hypertension management and it has also been associated to some antiviral activity which it is said could inhibit the replication of HIV-1 [64, 65]. The leaves are also important source of nutrients having 17 amino acids with adequate mineral composition like potassium, magnesium, phosphorus, calcium, sodium, zinc, manganese, and iron [65]. Participants in our study strongly believe that this herb helps them to control their elevated blood pressure. Some of the participants eat it before their clinic visits to maintain low blood pressure levels. A further study is required to evaluate whether hypertensive adults replace antihypertensive medication with *Nkaka* to lower their high blood pressure. This wild herb could be considered as an important component of dietary education for hypertension control in the area under study; however, further studies are also required to evaluate its effectiveness in lowering elevated blood pressure.

### Strengths and limitations

This study provides important in-depth cultural and social contexts to the implementation of public health interventions for adults with hypertension in a rural African setting. It provides a broad description of key determinants of physical activity and dietary considerations that strongly influence rural-based hypertension lifestyle interventions. As limitations we understand that in any focus group discussion, the inclusion of multiple people in one discussion does not guarantee full participation of everyone. For this reason we probe those participants who seemed to contribute less during the discussions in order to have the most from the group. In addition, all participants included in this study are part of an on-going cohort study on health in older population and have also been involved in other studies which may have influenced their perceptions and knowledge on the topic. This population might be more aware of health issues such as hypertension due to the regular study visits and clinic referrals, and may, therefore, not be representative of the general population. For individuals who declined to participate in our study, we did not ask reasons for non-participation, which creates a potential bias for non-response.

## Conclusion

This study provides useful insight for public health community-based interventions on physical activity and dietary sociocultural perceptions to be used when developing hypertension control interventions in rural African settings. We also highlight the need to design context specific interventions which make use of existing daily physical activity and dietary behaviour.

## Data Availability

The datasets used and/or analyzed during the current study include only transcripts of the interviews. Deidentified data may be made available from the corresponding author on reasonable request.

## Abbreviations

DASH: Dietary Approaches to Stop Hypertension
FGD: Focus Group Discussion
HDSS: Health and Demographic Surveillance System
HPT: Hypertension
LMIC: Low- and middle- income countries
MRC: Medical Research Center
PA: Physical Activity

## References

[1] World Health Organization. Global action plan for the prevention and control of noncommunicable diseases 2013–2020. World Health Organization; 2013.

[2] Zhou B, Bentham J, Di Cesare M, Bixby H, Danaei G, Cowan MJ, et al. Worldwide trends in blood pressure from 1975 to 2015: a pooled analysis of 1479 population-based measurement studies with 19· 1 million participants. Lancet. 2017;389(10064):37–55. 27863813 10.1016/S0140-6736(16)31919-5 PMC5220163

[3] Parati G, Lackland DT, Campbell NRC, Ojo Owolabi M, Bavuma C, Mamoun Beheiry H, et al. How to improve awareness, treatment, and Control of Hypertension in Africa, and how to reduce its consequences: a call to Action from the World Hypertension League. Hypertension. 2022;79(9):1949–61. 35638381 10.1161/HYPERTENSIONAHA.121.18884

[4] Mills KT, Stefanescu A, He J. The global epidemiology of hypertension. Nat Rev Nephrol. 2020;16(4):223–37. 32024986 10.1038/s41581-019-0244-2 PMC7998524

[5] Peltzer K, Phaswana-Mafuya N. Hypertension and associated factors in older adults in South Africa: cardiovascular topics. Cardiovasc J Afr. 2013;24(3):66–71. 10.5830/CVJA-2013-002 PMC3721893 23736129

[6] Lloyd-Sherlock P, Beard J, Minicuci N, Ebrahim S, Chatterji S. Hypertension among older adults in low-and middle-income countries: prevalence, awareness and control. Int J Epidemiol. 2014;43(1):116–28. 24505082 10.1093/ije/dyt215 PMC3937973

[7] Ibrahim MM, Damasceno A. Hypertension in developing countries. Lancet. 2012;380(9841):611–9. 22883510 10.1016/S0140-6736(12)60861-7

[8] Ntuli ST, Maimela E, Alberts M, Choma S, Dikotope S. Prevalence and associated risk factors of hypertension amongst adults in a rural community of Limpopo Province, South Africa. Afr J Prim Health Care Fam Med. 2015;7(1). 10.4102/phcfm.v7i1.847 PMC4685651 26842512

[9] Clark SJ, Gómez-Olivé FX, Houle B, Thorogood M, Klipstein-Grobusch K, Angotti N, et al. Cardiometabolic disease risk and HIV status in rural South Africa: establishing a baseline. BMC Public Health. 2015;15(1):1–9. 25885455 10.1186/s12889-015-1467-1 PMC4335669

[10] Ahaneku GI, Osuji CU, Anisiuba BC, Ikeh VO, Oguejiofor OC, Ahaneku JE. Evaluation of blood pressure and indices of obesity in a typical rural community in eastern Nigeria. Ann Afr Med. 2011;10(2). 10.4103/1596-3519.82076 21691018

[11] Hendriks ME, Wit FWNM, Roos MTL, Brewster LM, Akande TM, De Beer IH, et al. Hypertension in sub-saharan Africa: cross-sectional surveys in four rural and urban communities. PLoS ONE. 2012;7(3):e32638. 22427857 10.1371/journal.pone.0032638 PMC3299675

[12] Williams EA, Keenan KE, Ansong D, Simpson LM, Boakye I, Boaheng JM, et al. The burden and correlates of hypertension in rural Ghana: a cross-sectional study. Diabetes Metabolic Syndrome: Clin Res Reviews. 2013;7(3):123–8. 10.1016/j.dsx.2013.06.015 23953175

[13] Jardim TV, Reiger S, Abrahams-Gessel S, Gomez-Olive FX, Wagner RG, Wade A, et al. Hypertension management in a population of older adults in rural South Africa. J Hypertens. 2017;35(6):1283. 28441697 10.1097/HJH.0000000000001312 PMC5505070

[14] Abrahams-Gessel S, Gómez-Olivé FX, Tollman S, Wade AN, Du Toit JD, Ferro EG, et al. Improvements in hypertension control in the rural longitudinal HAALSI cohort of South African adults aged 40 and older, from 2014 to 2019. Am J Hypertens. 2023;36(6):324–32. 36857463 10.1093/ajh/hpad018 PMC10200554

[15] Cook I. Physical activity in rural South Africa-are current surveillance instruments yielding valid results? South Afr Med J. 2007;97(11):1072–3. 18250915

[16] Roos R, Myezwa H, van Aswegen H, Musenge E. Physical activity and risk factors screening for Ischaemic Heart Disease in South African Individuals Living with HIV.

[17] Temple NJ, Steyn NP, Fourie J, De Villiers A. Price and availability of healthy food: a study in rural South Africa. Nutrition. 2011;27(1):55–8. 20381314 10.1016/j.nut.2009.12.004

[18] Faber M, Wenhold F. Food intake and sources of food of poor households in rural areas of South Africa. Water use and nutrient content of crop and animal food products for improved household food security: A scoping study WRC Report no TT. 2012;537(12):24–57.

[19] Lloyd-Sherlock P, Gómez-Olivé FX, Ngobeni S, Wagner RG, Tollman S. Pensions and low sodium salt: a qualitative evaluation of a new strategy for managing hypertension in rural South Africa. Curr Aging Sci. 2018;11(2):140–6. 30019655 10.2174/1874609811666180718114250

[20] Dhar L, Earnest J, Ali M. A systematic review of factors influencing medication adherence to hypertension treatment in developing countries. Open J Epidemiol. 2017;7(03):211–50.

[21] Ferrans CE, Zerwic JJ, Wilbur JE, Larson JL. Conceptual model of health-related quality of life. J Nurs Scholarsh. 2005;37(4):336–42. 16396406 10.1111/j.1547-5069.2005.00058.x

[22] Shackleton CM, Shackleton SE, Buiten E, Bird N. The importance of dry woodlands and forests in rural livelihoods and poverty alleviation in South Africa. Policy Econ. 2007;9(5):558–77.

[23] Awumbila M. Drivers of migration and urbanization in Africa: key trends and issues. Int Migration. 2017;7(8).

[24] Bertram MY, Tollman S, Hofman KJ, Steyn K, Wentzel-Viljoen E. Reducing the sodium content of high-salt foods: effect on cardiovascular disease in South Africa. South Afr Med J. 2012;102(9):743–5. 10.7196/samj.5832 22958695

[25] He FJ, Li J, MacGregor GA. Effect of longer term modest salt reduction on blood pressure: Cochrane systematic review and meta-analysis of randomised trials. BMJ. 2013;346. 10.1136/bmj.f1325 23558162

[26] Lin JS, O’Connor EA, Evans CV, Senger CA, Rowland MG, Groom HC. Behavioral counseling to promote a healthy lifestyle for cardiovascular disease prevention in persons with cardiovascular risk factors. an updated systematic evidence review for the US Preventive Services Task Force; 2014.

[27] Kahn K, Collinson MA, Gómez-Olivé FX, Mokoena O, Twine R, Mee P, et al. Profile: Agincourt health and socio-demographic surveillance system. Int J Epidemiol. 2012;41(4):988–1001. 22933647 10.1093/ije/dys115 PMC3429877

[28] Gomez-Olive FX, Montana L, Wagner RG, Kabudula CW, Rohr JK, Kahn K, et al. Cohort profile: health and ageing in Africa: a longitudinal study of an indepth community in South Africa (HAALSI). Int J Epidemiol. 2018;47(3):689–j690. 29325152 10.1093/ije/dyx247 PMC6005147

[29] Stahl NA, King JR. Expanding approaches for research: understanding and using trustworthiness in qualitative research. J Dev Educ. 2020;44(1):26–8.

[30] Basit T. Manual or electronic? The role of coding in qualitative data analysis. Educational Res. 2003;45(2):143–54.

[31] Mattimoe R, Hayden MT, Murphy B, Ballantine J. Approaches to analysis of qualitative research data: A reflection on the manual and technological approaches. Accounting, Finance, & Governance Review. 2021;27(1).

[32] Maher C, Hadfield M, Hutchings M, De Eyto A. Ensuring rigor in qualitative data analysis: a design research approach to coding combining NVivo with traditional material methods. Int J Qual Methods. 2018;17(1):1609406918786362.

[33] Braun V, Clarke V. Thematic analysis. American Psychological Association; 2012.

[34] Hacking D, Haricharan HJ, Brittain K, Lau YK, Cassidy T, Heap M. Hypertension health promotion via text messaging at a community health center in South Africa: a mixed methods study. JMIR Mhealth Uhealth. 2016;4(1):e4569. 10.2196/mhealth.4569 PMC4807246 26964505

[35] Haricharan HJ, Heap M, Hacking D, Lau YK. Health promotion via SMS improves hypertension knowledge for deaf South africans. BMC Public Health. 2017;17(1):1–17. 28821288 10.1186/s12889-017-4619-7 PMC5563060

[36] Rampamba EM, Meyer JC, Helberg E, Godman B. Knowledge of hypertension and its management among hypertensive patients on chronic medicines at primary health care public sector facilities in South Africa; findings and implications. Expert Rev Cardiovasc Ther. 2017;15(8):639–47. 28712328 10.1080/14779072.2017.1356228

[37] Jongen VW, Lalla-Edward ST, Vos AG, Godijk NG, Tempelman H, Grobbee DE, et al. Hypertension in a rural community in South Africa: what they know, what they think they know and what they recommend. BMC Public Health. 2019;19(1):1–10. 30909905 10.1186/s12889-019-6642-3 PMC6434617

[38] Chimberengwa PT, Naidoo M. Group cooperative inquiry. Knowledge, attitudes and practices related to hypertension among residents of a disadvantaged rural community in southern Zimbabwe. PLoS ONE. 2019;14(6):e0215500. 31237883 10.1371/journal.pone.0215500 PMC6657811

[39] Agyei-Baffour P, Tetteh G, Quansah DY, Boateng D. Prevalence and knowledge of hypertension among people living in rural communities in Ghana: a mixed method study. Afr Health Sci. 2018;18(4):931–41. 30766557 10.4314/ahs.v18i4.12 PMC6354880

[40] De Wet H, Ramulondi M, Ngcobo ZN. The use of indigenous medicine for the treatment of hypertension by a rural community in northern Maputaland, South Africa. South Afr J Bot. 2016;103:78–88.

[41] Forman JP, Stampfer MJ, Curhan GC. Diet and lifestyle risk factors associated with incident hypertension in women. JAMA. 2009;302(4):401–11. 19622819 10.1001/jama.2009.1060 PMC2803081

[42] Carey RM, Whelton PK. Committee* 2017 ACC/AHA Hypertension Guideline Writing. Prevention, detection, evaluation, and management of high blood pressure in adults: synopsis of the 2017 American College of Cardiology/American Heart Association Hypertension Guideline. Ann Intern Med. 2018;168(5):351–8. 29357392 10.7326/M17-3203

[43] Aronow WS. Association of obesity with hypertension. Ann Transl Med. 2017;5(17). 10.21037/atm.2017.06.69 PMC5599277 28936444

[44] Kaneva AM, Bojko ER. Sex differences in the association between obesity and hypertension. Arch Physiol Biochem. 2023;129(3):682–9. 33476209 10.1080/13813455.2020.1861027

[45] Oyewumi O. African gender studies: a reader. Springer; 2016.

[46] Biernat E, Piątkowska M. Leisure time physical activity among employed and unemployed women in Poland. Hong Kong J Occup Therapy. 2017;29(1):47–54. 10.1016/j.hkjot.2017.04.001 PMC6092003 30186072

[47] Francis D, Webster E. Poverty and inequality in South Africa: critical reflections. Dev South Afr. 2019;36(6):788–802.

[48] Appel LJ, Moore TJ, Obarzanek E, Vollmer WM, Svetkey LP, Sacks FM, et al. A clinical trial of the effects of dietary patterns on blood pressure. N Engl J Med. 1997;336(16):1117–24. 9099655 10.1056/NEJM199704173361601

[49] Steinberg D, Bennett GG, Svetkey L. The DASH diet, 20 years later. JAMA. 2017;317(15):1529–30. 28278326 10.1001/jama.2017.1628 PMC5509411

[50] Santos PCJL, Krieger JE, Pereira AC. Renin–angiotensin system, hypertension, and chronic kidney disease: pharmacogenetic implications. J Pharmacol Sci. 2012;120(2):77–88. 23079502 10.1254/jphs.12r03cr

[51] Desalegn BB, Lambert C, Riedel S, Negese T, Biesalski HK. Ethiopian orthodox fasting and lactating mothers: longitudinal study on dietary pattern and nutritional status in rural Tigray, Ethiopia. Int J Environ Res Public Health. 2018;15(8):1767. 30126089 10.3390/ijerph15081767 PMC6121597

[52] Adeyeye SAO. The role of food processing and appropriate storage technologies in ensuring food security and food availability in Africa. Nutr Food Sci. 2017;47(1):122–39.

[53] Labadarios D, Mchiza ZJR, Steyn NP, Gericke G, Maunder EMW, Davids YD, et al. Food security in South Africa: a review of national surveys. Bull World Health Organ. 2011;89(12):891–9. 22271946 10.2471/BLT.11.089243 PMC3260897

[54] Statistics South Africa. www.statssa.gov.za. 2023 [cited 2023 May 16]. Beyond unemployment - Time-related underemployment in the SA labour market. www.statssa.gov.za.

[55] Charlton KE, Steyn K, Levitt NS, Peer N, Jonathan D, Gogela T, et al. A food-based dietary strategy lowers blood pressure in a low socio-economic setting: a randomised study in South Africa. Public Health Nutr. 2008;11(12):1397–406. 18752692 10.1017/S136898000800342X

[56] Ware LJ, Charlton K, Schutte AE, Cockeran M, Naidoo N, Kowal P, Nutrition. Metabolism Cardiovasc Dis. 2017;27(9):784–91. 10.1016/j.numecd.2017.06.017 28800936

[57] Charlton KE, Corso B, Ware L, Schutte AE, Wepener L, Minicuci N, et al. Effect of South Africa’s interim mandatory salt reduction programme on urinary sodium excretion and blood pressure. Prev Med Rep. 2021;23:101469. 34381665 10.1016/j.pmedr.2021.101469 PMC8333157

[58] Du Toit JD, Kapaon D, Crowther NJ, Abrahams-Gessel S, Fabian J, Kabudula CW, et al. Estimating population level 24-h sodium excretion using spot urine samples in older adults in rural South Africa. J Hypertens. 2023;41(2):280. 36583353 10.1097/HJH.0000000000003327 PMC10198169

[59] Organization WH. Guideline: potassium intake for adults and children. World Health Organization; 2012. 23617019

[60] Guwatudde D, Nankya-Mutyoba J, Kalyesubula R, Laurence C, Adebamowo C, Ajayi I, et al. The burden of hypertension in sub-saharan Africa: a four-country cross sectional study. BMC Public Health. 2015;15(1):1–8. 26637309 10.1186/s12889-015-2546-z PMC4670543

[61] Chobanian AV. Guidelines for the management of hypertension. Med Clin. 2017;101(1):219–27. 10.1016/j.mcna.2016.08.016 27884231

[62] World Health Organization. Guideline for the pharmacological treatment of hypertension in adults. World Health Organization; 2021. 34495610

[63] Tussing-Humphreys L, Thomson JL, Mayo T, Edmond E. Peer reviewed: a church-based diet and physical activity intervention for rural, lower Mississippi Delta African American adults: Delta body and soul effectiveness study, 2010–2011. Prev Chronic Dis. 2013;10. 10.5888/pcd10.120286 PMC3682809 23742940

[64] Jandari S, Ghavami A, Ziaei R, Nattagh-Eshtivani E, Rezaei Kelishadi M, Sharifi S, et al. Effects of Momordica charantia L on blood pressure: a systematic review and meta-analysis of randomized clinical trials. Int J Food Prop. 2020;23(1):1913–24.

[65] Thakur GS, Bag M, Sanodiya BS, Bhadauriya P, Debnath M, Prasad G, et al. Momordica balsamina: a medicinal and neutraceutical plant for health care management. Curr Pharm Biotechnol. 2009;10(7):667–82. 19751180 10.2174/138920109789542066

